# Dietary Supplement, Containing the Dry Extract of *Curcumin*, *Emblica* and *Cassia*, Counteracts Intestinal Inflammation and Enteric Dysmotility Associated with Obesity

**DOI:** 10.3390/metabo13030410

**Published:** 2023-03-09

**Authors:** Vanessa D’Antongiovanni, Matteo Fornai, Laura Benvenuti, Clelia Di Salvo, Carolina Pellegrini, Federica Cappelli, Stefano Masi, Luca Antonioli

**Affiliations:** Department of Clinical and Experimental Medicine, University of Pisa, 56126 Pisa, Italy

**Keywords:** natural compound, obesity, high-fat diet, *Curcumin*, *Emblica*, *Cassia*, gut dysmotility, enteric inflammation, intestinal barrier alterations, dietary supplements

## Abstract

Intestinal epithelial barrier (IEB) impairment and enteric inflammation are involved in the onset of obesity and gut-related dysmotility. Dietary supplementation with natural plant extracts represents a useful strategy for the management of body weight gain and systemic inflammation associated with obesity. Here, we evaluate the efficacy of a food supplement containing the dry extract of *Curcumin*, *Emblica* and *Cassia* in counteracting enteric inflammation and motor abnormalities in a mouse model of obesity, induced by a high-fat diet (HFD). Male C57BL/6 mice, fed with standard diet (SD) or HFD, were treated with a natural mixture (*Curcumin*, *Emblica* and *Cassia*). After 8 weeks, body weight, BMI, liver and spleen weight, along with metabolic parameters and colonic motor activity were evaluated. Additionally, plasma LBP, fecal calprotectin, colonic levels of MPO and IL-1β, as well as the expression of occludin, TLR-4, MYD88 and NF-κB were investigated. Plant-based food supplement administration (1) counteracted the increase in body weight, BMI and metabolic parameters, along with a reduction in spleen and liver weight; (2) showed strengthening effects on the IEB integrity; and (3) reduced enteric inflammation and oxidative stress, as well as ameliorated the colonic contractile dysfunctions. Natural mixture administration reduced intestinal inflammation and counteracted the intestinal motor dysfunction associated with obesity.

## 1. Introduction

Obesity is a pathological condition characterized as excessive fat accumulation, resulting from an imbalance between energy intake and its consumption [1]. At present, obesity represents a public health problem for its involvement in multiple chronic diseases, such as type 2 diabetes, hypertension and cardiovascular diseases [1,2]. In addition, obesity is often associated with digestive disturbances, including infrequent bowel movements and constipation, which negatively impact the patients’ quality of life and complicate their clinical management [3]. 

Several lines of evidence well demonstrated that the presence of a low-grade systemic inflammation, commonly defined as “meta-inflammation”, resulting from increased pro-inflammatory cytokine and chemokine secretion from adipocytes and adipose tissue-associated macrophages, seems to contribute to the impairment of insulin signaling, immune function and lipid metabolism, as well as a remarkable morphofunctional remodelling of the enteric neuromuscular compartment, resulting in bowel motor dysfunctions [2,4,5]. In particular, pre-clinical studies on high-fat diet (HFD)-fed mice showed that alterations in intestinal microbiota composition and the presence of an increased intestinal permeability represent among the main factors influencing early inflammatory events associated with obesity and metabolic dysfunction [6,7,8]. Indeed, the presence of an imbalanced gut microbiota composition has been reported to elicit fluctuations in the intestinal epithelial barrier (IEB) integrity, thus facilitating the translocation of immunogenic products (i.e., lipopolysaccharide (LPS), whole bacteria and other toxins) into the mucosa and the bloodstream, triggering the onset and maintenance of a meta-inflammation that is typically observed in obese patients [9,10,11]. Of note, the chronicization of such inflammatory conditions has an impact on the physiological functions of different organs, thus contributing to the development of several comorbidities associated with obesity [5].

At present, the pharmacological tools aimed at managing obesity and related comorbidities are not satisfactory in terms of efficacy, safety and long-lasting weight loss. In this regard, pre-clinical and clinical studies have focused their attention on the potential beneficial effects of natural derivates, such as crude extracts and products isolated from plants, as a viable way to manage obesity and related disorders. In recent years, several in vivo studies performed on a murine model of diet-induced obesity showed that dietary supplementation with natural drugs, such as *Curcumin*, *Emblica* and *Cassia*, might exert anti-obesity activity, counteracting body weight gain and systemic inflammation associated with obesity [12,13,14]. Of note, current studies on the putative beneficial effects of natural products supplementation on intestinal functional disorders and enteric inflammation associated with obesity are lacking. Therefore, the present research project was designed to assess the effect of a dietary supplement, containing the dry extract of *Curcumin*, *Emblica* and *Cassia*, in preventing and counteracting the intestinal motor dysfunction and the onset of enteric inflammation in a mouse model of diet-induced obesity.

## 2. Experimental Design

### 2.1. Animals 

Five-week-old C57BL/6 male mice (body weight: 20–22 g) were provided by ENVIGO S.r.l (San Pietro al Natisone UD, Italy) and used during all the study’s procedures. Animals were divided into groups of five in cages in a climate-controlled environment, with a 12-h light cycle, at 22–24 °C and 50–60% humidity, and they were not used for at least a week. All experimental procedures involving mice were carried out following the Declaration of Helsinki, the EU Regulation 2010/63/EU for animal experimentation, and the European Community Council Directive 86/609, as well as the International Medical Association’s Code of Ethics. The University of Pisa’s Ethics Council for Animal Experimentation and the Italian Ministry of Health have both given their approval for the studies (Authorization No. 987/2020-PR). All efforts to reduce and minimize the number of animals and their suffering were carried out. Animal studies are reported in compliance with the ARRIVE [15]. Animals were distributed randomly across groups to create groups of the same size.

### 2.2. Animal Model of Diet-Induced Obesity and Experimental Design

To induce the obesity, for 8 weeks, mice were fed with HFD (60% calories from fat) or standard diet (SD, 18% calories from fat). HFD diet included 18.3% kcal from proteins, 21.4% kcal from carbohydrates and 60.8% kcal from fat, whereas SD provided 24% kcal from proteins, 18% kcal from fat and 58% kcal from carbohydrates. Animals were randomly divided into ten groups, each composed of ten mice as follow: SD or HFD plus vehicle for a total of 8 weeks (treatment 4 + 4 weeks), SD or HFD treated with food supplement containing three plants (*Curcuma longa*—46% *w*/*v*; *Cassia mimosoides* var. *Nomame*—15.5% *w/v;* and *Phyllanthus emblica*—15.5% *w*/*v*) starting from the fifth week (treatment 4 + 4 weeks), and SD or HFD plus each individual component of food supplement starting from the fifth week (treatment 4 + 4 weeks). Dietary supplement and each plant contained in the supplement were administered via oral gavage at the following doses: dietary supplement—107 mg/Kg/die; *Curcuma* longa (*Curcuma* L.)—49 mg/Kg/die; *Cassia mimosoides* var. *Nomame* (*Cassia* M.)—16 mg/Kg/die; and *Phyllanthus emblica* (*P. emblica*)—16 mg/Kg/die. The natural mixture and each individual components were provided by Snep S.p.A, Pisa, Italy. Starting from the first day of the study, body weight was recorded once a week. At the end of the study, the animals were anaesthetized and sacrificed. Blood samples and tissue samples were collected and then stored at −80 °C for the following analyses.

### 2.3. Measurement of Body Mass Index and Evaluation of Metabolic Parameters 

Body mass index (BMI) was calculated as body weight (g) divided by body length (mm) squared (BMI = body weight/body length^2^), as described by Smemo et al. [16].

Blood samples were obtained from the tail vein the day of sacrifice, after an overnight fast. Glycated hemoglobin (HbA1c), cholesterol and triglycerides were evaluated using the Multicare Insensor (BSI Srl; Arezzo, Italy), following manufacturer’s instructions [17]. 

### 2.4. Recording of Colonic Contractile Activity

The contractile activity of colonic longitudinal smooth muscle preparations was recorded, as previously reported [18], with minor changes. Following sacrifice, by making an incision above the anal end, the colon was promptly removed and put in Krebs solution. Segments of colon were opened along the mesenteric insertion and mucosal/submucosal layers were removed. Colonic samples were slitted along the longitudinal axis into slices of around 4-mm in width and 10-mm in length. The preparations were set up in organ baths containing Krebs solution at 37 °C, bubbled with 5% CO_2_ and 95% O_2_, and connected to isometric transducers (constant load = 0.5 g). BIOPAC MP150 (Biomedica Mangoni; Pisa, Italy) registered the mechanical activity. The Krebs solution was prepared as indicated in the following, with values represented as mM: KCl—4.7; NaCl—113; KH_2_PO_4_—1.2; CaCl_2_—2.5; MgSO_4_—1.2; NaHCO_3_—25; glucose—11.5 (pH 7.4 ± 0.1). Each preparation was allowed to equilibrate for at least 30 min, with washings every ten minutes. To deliver electrical stimulation using a BM-ST6 stimulator (Biomedica Mangoni, Pisa, Italy), a pair of coaxial platinum electrodes were placed 10 mm from the longitudinal axis of each preparation. After the equilibration time, electrical stimuli were repeatedly applied to each preparation, and experiments began when repeatable responses were obtained (generally after three stimulations). Thanks to previous experiments, the appropriate electrical stimulation frequency (10 Hz) and exogenous substance P concentration were selected. The neurogenic NK1 contractions were recorded in colonic specimens maintained in Krebs solution, containing N-ω-nitro-L-arginine methylester (L-NAME, nitric oxide synthase inhibitor, 100 µM), guanethidine (adrenergic blocker, 10 µM), 5-fluoro-3-[2-[4-methoxy-4-[[(R)-phenylsulphinyl]methyl]-1-piperidinyl]ethyl]-1H-indole (GR159897, NK2 receptor antagonist, 1 µM), (R)-[[(2-phenyl-4-quinolinyl)carbonyl]amino]-methyl ester benzeneacetic acid (SB218795, NK3 receptor antagonist, 1 µM) and atropine sulphate (muscarinic receptor antagonist, 1 µM), to assess neurogenic contraction, whereas the myogenic activity was detected maintaining the specimens in Krebs solution with the addition of tetrodotoxin (TTX, 1 µM) and stimulating with exogenous substance P (SP, 1 µM).

### 2.5. Evaluation of Plasma Lipopolysaccharide Binding Protein 

Lipopolysaccharide binding protein (LPB) level in plasma was measured by ELISA (Prodotti Gianni; Milan, Italy), as previously described [10,19]. For the procedure, blood samples were centrifuged for 5 min at 4000 rpm at 2–8 °C and, after the centrifugation, supernatants were collected. Aliquots (100 µL) were used for the procedure. LBP levels were expressed as ng/mL of plasma.

### 2.6. Quantification of Plasma Myeloperoxidase and Colonic Interleukin-1β Levels

Plasmatic myeloperoxidase (MPO) levels, positively linked to obesity-related inflammation and insulin resistance induced by the diet [20,21], was measured by ELISA (Prodotti Gianni; Milan, Italy), as previously described [22]. Blood samples were centrifuged for 5 min at 4000 rpm at 2–8 °C and, after the centrifugation, supernatants were collected. Aliquots of 100 μL were used for the assays. MPO levels were expressed as ng/mL of plasma. Tissue IL-1β levels were quantified, as previously described [23], using a commercial ELISA Kit (Abcam; Cambridge, United Kingdom). Colon tissues, previously collected and stored at −80° C, were briefly thawed, weighed, and homogenized in PBS (0.4 mL/20 mg of tissue) at 4 °C, and centrifuged for 5 min at 10,000× *g*. Aliquots of 100 µL were used to conduct the experiment. IL-1β levels were indicated as picograms per milligram (pg/mg) of protein.

### 2.7. Assay of Faecal Calprotectin 

Calprotectin, a calcium binding protein of neutrophil granulocytes regarded as an index of neutrophil infiltration in the intestinal mucosa, was assessed in faecal samples. Briefly, freeze-dried faecal pellets were reconstituted in 1 mL PBS, along with 50 μL of 1% (wt/vol) ascorbic acid (Sigma; St Louis, MO, USA). Samples were then homogenized for ten minutes (4 °C). Homogenates were diluted with 2 mL lysis buffer (0.1% sodium dodecylsulfate, 0.5% sodium deoxycholate, 0.02% sodium azide, 5 mM disodium ethylenediaminetetraacetic acid, and 1× Halt protease/phosphatase inhibitor cocktail [Thermo Fisher Scientific Inc.; Waltham, MA, USA] in PBS). Homogenates were further homogenized for 30 s and centrifuged (5800× *g*, 10 min, 4 °C), and supernatants were frozen in liquid nitrogen and stocked at −80 °C. Faecal calprotectin levels were determined using mouse calprotectin enzyme-linked immunosorbent assay kit and analyzed in accordance with the manufacturer’s instructions.

### 2.8. Western Blot Assays

Colonic tissues were lysed as previously reported [24,25]. Briefly, tissues were weighed and then homogenized in lysis buffer (50 mg in 400 µL), using a polytron homogenizer (QIAGEN; Milan, Italy). Homogenates were centrifuged at 12,000 rpm for 15 min at 4 °C, and supernatants were then separated from pellets and conserved at −80 °C. Bradford assay was performed to quantify total proteins. Subsequently, proteins were separated onto a pre-cast 4–20% polyacrylamide gel (Mini-PROTEAN TGX gel, Biorad, Hercules, CA, United States) and transferred to PVDF membranes (Trans-Blot TurboTM PVDF Transfer packs, Biorad, Hercules, CA, USA). Membranes were blocked with 3% BSA diluted in Tris-buffered saline (TBS, 20 mM Tris-HCl, PH 7.5, 150 mM NaCl) with 0.1% Tween 20. Primary antibodies against glyceraldehyde 3-phosphate dehydrogenase (GAPDH, 5174S, Cell Signaling, Massachusetts, USA), occludin (ab167161, Abcam, Cambridge, UK), toll-like receptor (TLR)-4 (ab22048, Abcam, Cambridge, UK), nuclear factor kB p65 (NF-κB-p65, sc-8008, Santa Cruz, Dallas, USA) and myeloid-differentiation primary response-gene 88 (MyD88, sc-136970, Santa Cruz, Dallas, USA) were used. Secondary antibodies were bought from Abcam (anti-mouse ab97040 and anti-rabbit ab6721). Protein bands were revealed with ECL reagents (Clarity Western ECL Blotting Substrate, Biorad, Hercules, CA, USA). iBright Analysis software was used to perform the densitometry analysis.

### 2.9. Statistical Analysis

The statistical analysis was performed only for studies where each group size was at least n = 6. In particular, the results are presented as mean ± standard error of the mean (S.E.M.). Two-way ANOVA or one-way ANOVA was used to assess statistical significance, followed by Tukey’s post hoc tests. Significant differences were obtained with *p* values < 0.05. All statistical procedures were performed by two different operators, blinded to the treatment, using GraphPad Prism 7.0 software (GraphPad Prism; San Diego, CA, USA).

## 3. Results 

### 3.1. Plant-Based Food Supplement Counteracts the Body Weight Gain and BMI in Obese Mice

**Figure 1:**
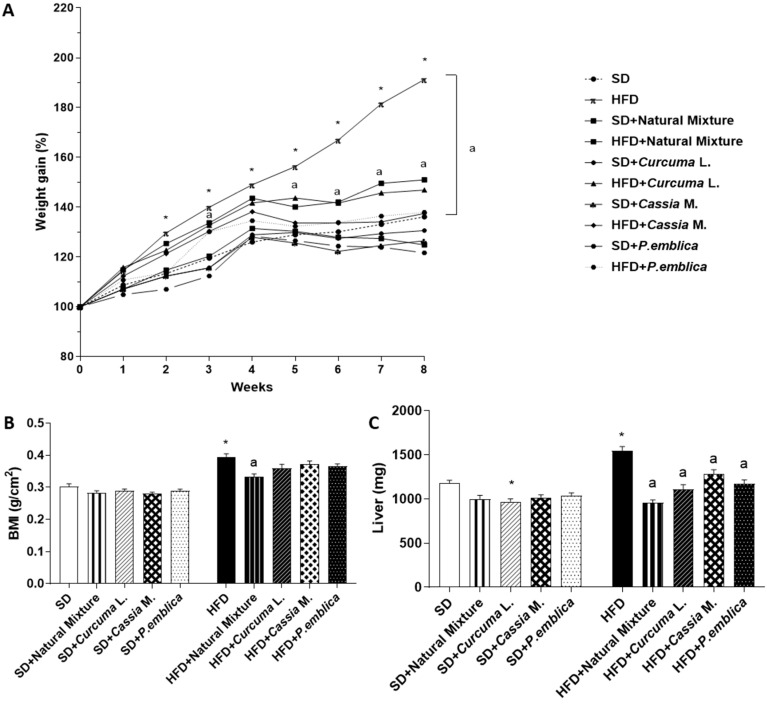

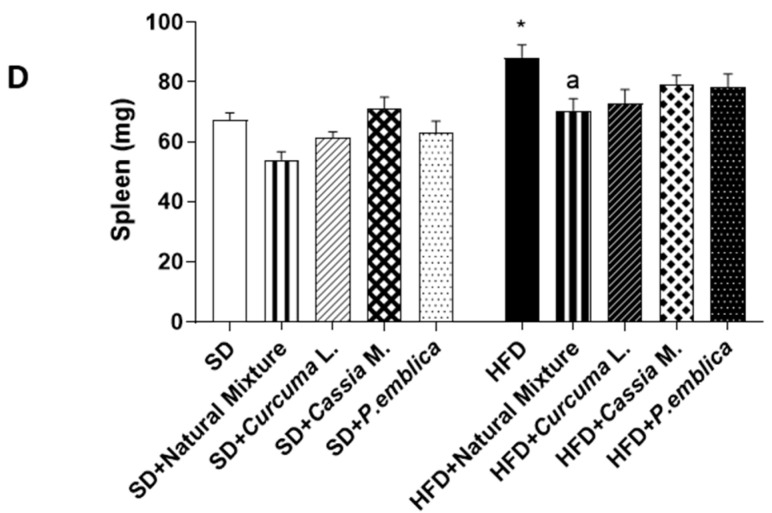
Effects of natural mixture, *Curcuma* L., *Cassia* M. and *P. emblica* on body weight (**A**), BMI (**B**), liver weight (**C**) and spleen weight (**D**) in HFD- and SD-fed mice. Each column shows the mean ± SEM (*n* = 10). Two-way and one-way ANOVA, and Tukey post hoc test results are as follows: *—*p* < 0.05 for significant difference vs. the SD group, and ^a^—*p* < 0.05 for significant difference vs. the HFD group. Abbreviations: BMI—body mass index; HFD—high fat diet; SD—standard diet.

HFD mice showed a significant increase in body weight compared to SD animals (Figure 1A). Dietary supplementation with either the natural mixture or each individual component (*Curcuma* L., *Cassia* M. and *P. emblica*) significantly counteracted the body weight gain in HFD-fed mice (Figure 1A). In SD-fed mice, neither the dietary supplement nor the administration of each individual component influenced body weight gain throughout the 8 weeks, as compared with SD animals (Figure 1A). At the end of week 8, BMI was significantly increased in HFD mice compared to SD mice (Figure 1B). Supplementation with the natural mixture significantly counteracted such an increase in HFD-fed mice, while each individual component slightly affected this parameter (Figure 1B). Plant-based food supplement administration and each individual component did not affect BMI in SD-fed mice (Figure 1B).

### 3.2. Dietary Supplementation with Curcumin, Emblica and Cassia Reduces Spleen and Liver Weight 

Mice fed with HFD for 8 weeks showed a significant increase in spleen and liver weight compared to SD mice (Figure 1C,D). The weight of the spleen and liver were significantly reduced in HFD-fed mice administered with dietary supplement for 8 weeks (Figure 1C,D). Of note, supplementation with *Curcuma* L., *Cassia* M. and *P. emblica* significantly reduced liver weight, but not spleen weight in HFD mice (Figure 1C,D). No significant differences were observed for spleen and liver weight between SD group and the natural mixture treated-SD mice (Figure 1C,D).

### 3.3. Plant-Based Food Supplement Ameliorated Metabolic Parameters in HFD Mice

**Figure 2:**
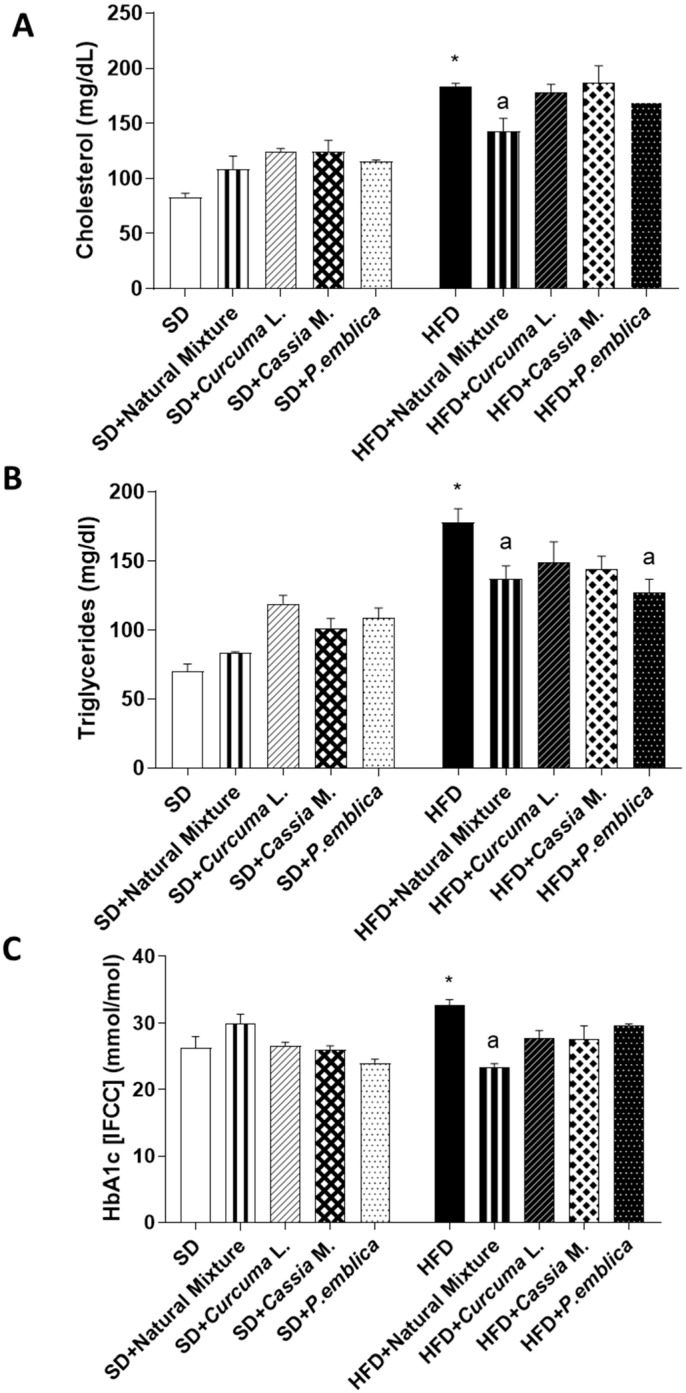
Effects of natural mixture, *Curcuma* L., *Cassia* M. and *P. emblica* on cholesterol (**A**), triglycerides (**B**) and glycated hemoglobin (**C**) in HFD- and SD-fed mice. Each column shows the mean ± SEM (*n* = 6). One-way ANOVA and Tukey post hoc test results are as follows: *—*p* < 0.05 for significant difference vs. the SD group, and ^a^—*p* < 0.05 for significant difference vs. the HFD group. Abbreviations: HbA1c—glycated haemoglobin; HFD—high fat diet; SD—standard diet.

After 8 weeks of HFD diet, mice showed a significant increase in HbA1c, cholesterol and triglycerides levels compared to SD mice (Figure 2A–C). The supplementation with the dietary supplement for 8 weeks determined a significant reduction in all the assayed parameters in HFD animals, while there was no significant difference in metabolic parameter levels between the SD group and the SD mice treated with the natural mixture (Figure 2A–C). 

### 3.4. Plant-Based Food Supplement Ameliorates the Intestinal Barrier Integrity 

**Figure 3:**
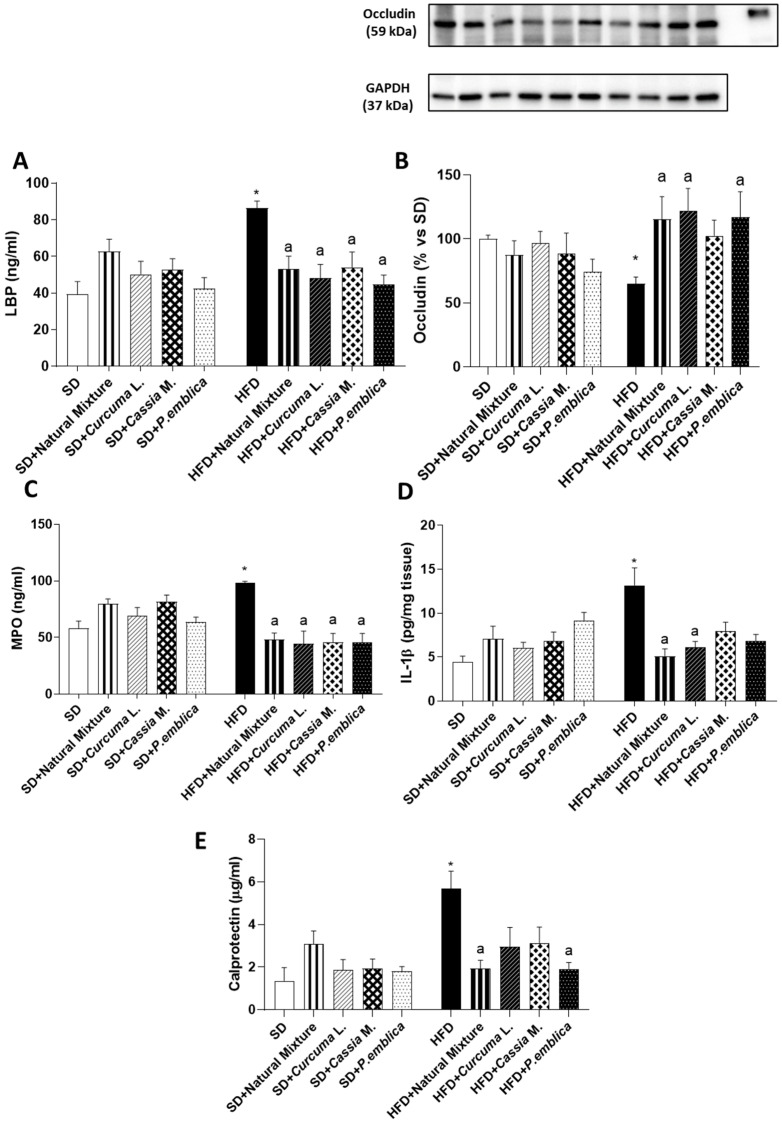
(**A**) Circulating LBP in SD and HFD mice treated with natural mixture, *Curcuma* L., *Cassia* M. and *P. emblica*. (**B**) Densitometric analysis and representative blots of occludin expression in colonic tissues from HFD- and SD-fed mice treated with natural mixtures and each single component. Levels of (**C**) plasmatic MPO and (**D**) colonic IL-β from SD and HFD mice treated with natural mixture and each single component. (**E**) Calprotectin levels in fecal samples from SD and HFD mice treated with natural mixture, *Curcuma* L., *Cassia* M. and *P. emblica*. Each column shows the mean ± SEM (*n* = 6). One-way ANOVA and Tukey post hoc test results are as follows: *—*p* < 0.05 for significant difference vs. the SD group, and ^a^—*p* < 0.05 for significant difference vs. the HFD group. Abbreviations: HFD—high fat diet; IL-1β—interleukin-1β; LBP—lipopolysaccharide-binding protein; MPO—Myeloperoxidase; SD—standard diet; GAPDH—Glyceraldehyde 3-phosphate dehydrogenase.

Mice fed with HFD for 8 weeks displayed a significant increase in LBP plasma levels, along with a reduction in colonic occludin levels, as compared with SD animals (Figure 3A,B). Food supplementation with natural mixture or with *Curcuma* L. and *P. emblica* alone determined a normalization of LBP and occludin expression (Figure 3A,B). Plant-based food supplement administration and each individual component did not affect plasma LBP levels and the expression of occludin in SD-fed mice (Figure 3A,B).

### 3.5. Dietary Supplementation with Curcumin, Emblica and Cassia Reduces Plasmatic MPO and Tissutal IL-1β Levels in HFD Mice

After 8 weeks of HFD diet, mice showed an increase in circulating MPO levels, as compared with SD animals (Figure 3C). Dietary supplementation with the natural mixture or each individual component significantly reduced plasmatic MPO levels in HFD mice (Figure 3C). 

IL-1β levels also increased in colonic tissues from HFD mice compared to SD mice (Figure 3D). Plant-based food supplement administration and *Curcuma* L. significantly counteracted such an increase in HFD-fed mice (Figure 3D). Of note, each individual component did not affect the assayed parameters in SD-fed mice (Figure 3C,D).

### 3.6. Plant-Based Food Supplement Reduced Faecal Calprotectin Levels in Obese Mice 

HFD resulted in a significant increase in faecal calprotectin levels, as compared with SD (Figure 3E). Supplementation with the natural mixture and *P. emblica* alone led to a significant reduction in faecal calprotectin in HFD mice, as compared to control HFD mice (Figure 3E). Of note, food supplement administration and each individual component did not affect calprotectin values in SD-fed mice (Figure 3E).

### 3.7. Dietary Supplementation with Curcumin, Emblica and Cassia Reduces Colonic Expression of TLR-4, MyD88 and NF-κB

**Figure 4:**
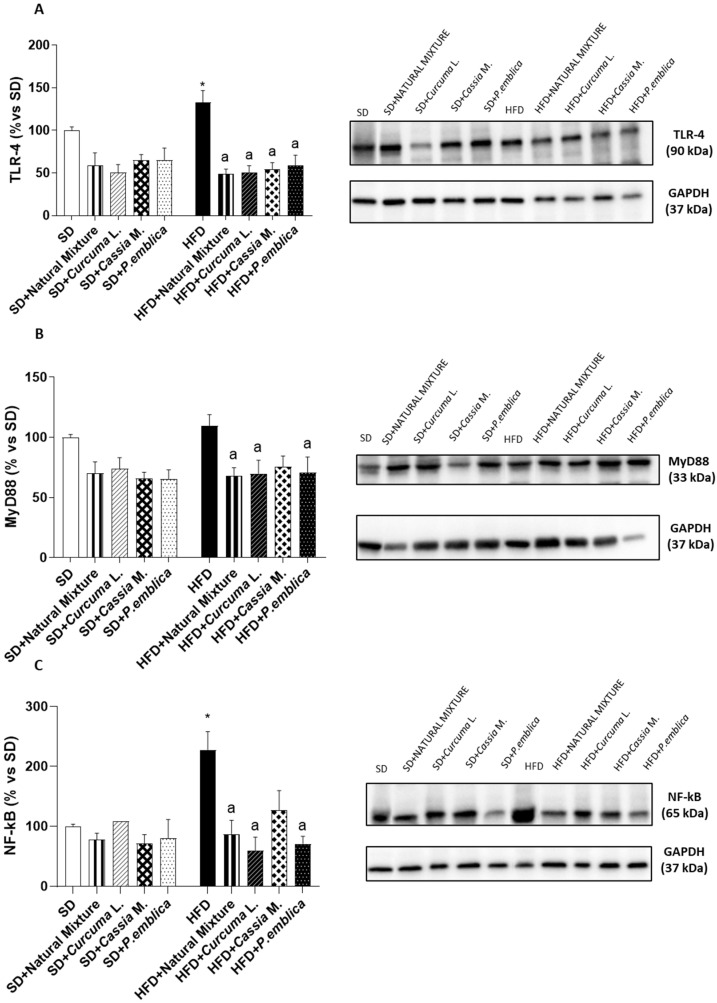
Densitometric analysis and representative blots of the expression of (**A**) TLR-4, (**B**) MyD88 and (**C**) NF-kB in colonic tissues from HFD- or SD-fed mice treated with natural mixtures or with the single components. Each column shows the mean ± SEM (*n* = 6). One-way ANOVA are Tukey post hoc test results are as follows: *—*p* < 0.05 for significant difference vs. the SD group, and ^a^—*p* < 0.05 for significant difference vs. the HFD group. Abbreviations: HFD—high fat diet; MyD88—myeloid-differentiation primary response-gene 88; NF-κB—nuclear factor kB; SD—standard diet; TLR-4—toll-like receptor 4; GAPDH—Glyceraldehyde 3-phosphate dehydrogenase.

Colonic tissues from HFD animals showed an increase in TLR-4 expression, compared to SD mice (Figure 4A). Supplementation with the natural mixture or each individual component significantly reduced TLR-4 expression in obese mice (Figure 4A). In addition, in obese mice, the colonic expression of MyD88 and NF-kB increased, as compared with SD group (Figure 4B,C). Treatment with food supplement or with *Curcuma* L. or *P. emblica* alone determined a significant reduction in MyD88 and NF-kB expression in HFD mice (Figure 4B,C). Of note, food supplement administration and each individual component did not affect the above parameters in SD-fed mice (Figure 4A–C).

### 3.8. Plant-Based Food Supplement Counteracted Colonic Dysmotility in Obese Mice 

During the equilibration period, some colonic preparations developed spontaneous contraction. Such contractile activity of low amplitude remained stable during the experiment and did not interfere with the motor responses evoked by electric stimuli (ES). Electrically elicited responses consisted of phasic contractions which were, in some cases, followed by after-contractions of variable amplitude. 

**Figure 5:**
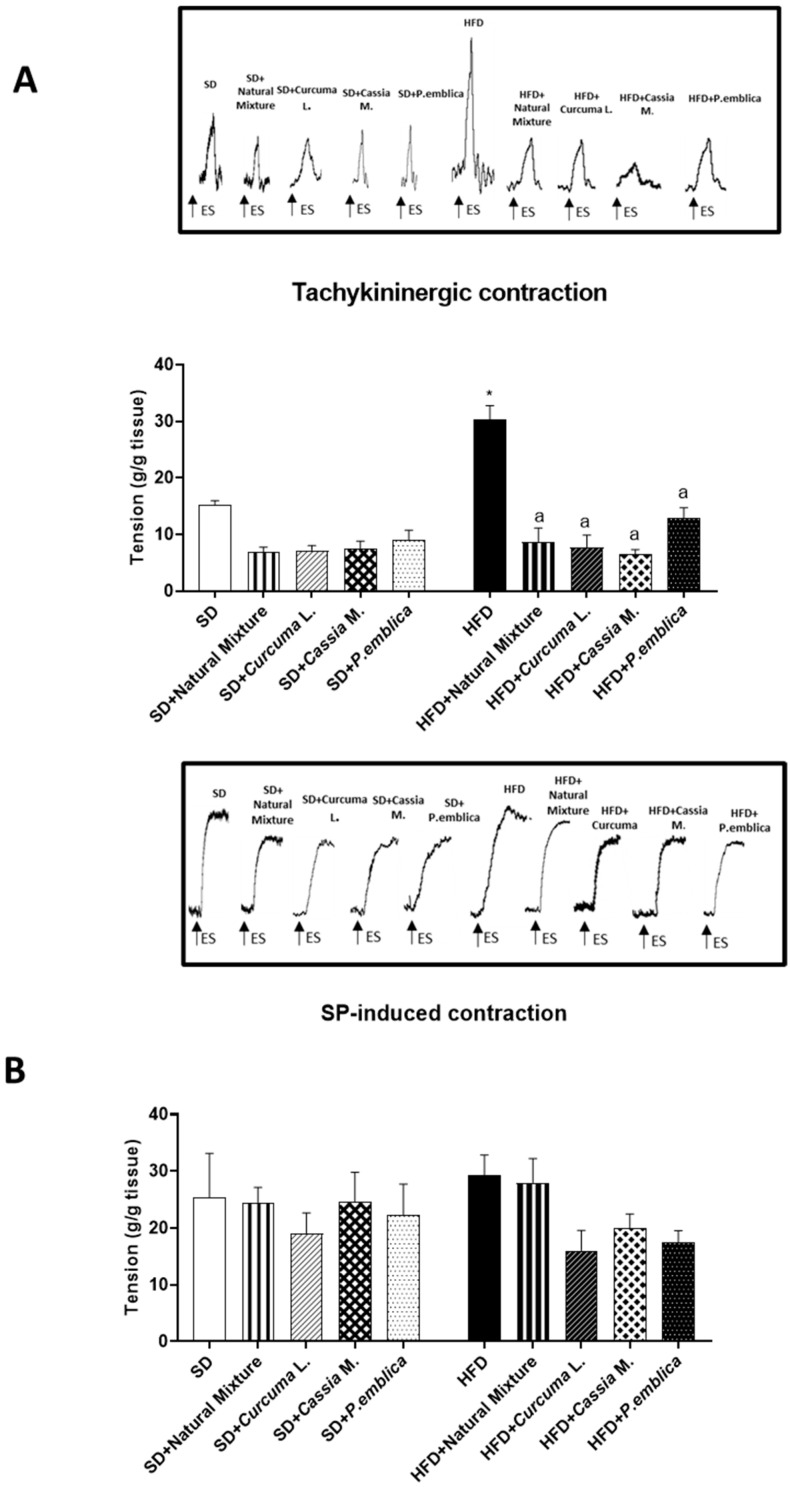
Effect of natural mixture on in vitro colonic contractile responses. (**A**) Tachykininergic contractions mediated by NK1 of colonic longitudinal smooth muscle preparations taken from HFD- and SD-fed mice treated with natural mixture, *Curcuma* L., *Cassia* M. and *P. emblica*. (**B**) Contractions elicited by exogenous SP (1 μM) in colonic preparations taken from mice treated with natural mixture, *Curcuma* L., *Cassia* M. and *P. emblica*. Each column shows the mean ± S.E.M. (*n* = 6). One-way ANOVA and Tukey post hoc test results are as follows: *—*p <* 0.05 for significant difference vs. the SD group; ^a^—*p <* 0.05 for significant difference vs. the HFD group. Abbreviations: HFD— high fat diet; SD—standard diet; SP—substance P.

Colonic preparations from mice fed with HFD for 8 weeks, maintained in Krebs solution with 10 μM guanethidine, 100 μM L-NAME, 1 μM atropine, 1 μM GR159897 and 1 μM SB218795, showed a significant increment of electrically evoked NK1-mediated tachykininergic contractions, compared to SD animals (Figure 5A). Dietary supplementation with the natural mixture or each individual component determined a normalization of colonic contractile responses, counteracting the overactivity of the tachykininergic system (Figure 5A). 

The stimulation, induced by exogenous substance P (SP) of colonic preparations from SD and HFD mice, untreated and treated with natural mixture, elicited contractions of similar magnitude, suggesting no alterations in SP-induced myogenic contractions (Figure 5B). 

## 4. Discussion and Conclusions

Obese patients often experience—alongside several associated comorbidities, including cardiovascular disease, non-alcoholic fatty liver disease, type 2 diabetes and cancer— gastrointestinal disturbances, including constipation and delayed gastric emptying [5,26,27]. In this context, gut microbiota alterations, impairments of IEB integrity and enteric inflammation have been identified as key players in the onset of obesity and gut-related dysmotility [6,7,8]. In the last few years, several lines of evidence have suggested that dietary supplementation with natural plant extracts represents a useful strategy for the management of body weight gain and systemic inflammation related to obesity. 

On these bases, in the present study, we evaluated the efficacy of a food supplement, containing the dry extract of *Curcumin*, *Emblica* and *Cassia*, in counteracting the onset of enteric inflammation and the development of motor abnormalities in a mouse model of diet-induced obesity. In particular, our experiments pointed out three major novel findings. The natural mixture supplementation (1) counteracted the increase in body weight, BMI and metabolic parameters, along with a reduction in spleen and liver weight; (2) exerts strengthening effects on the IEB integrity; and (3) reduces enteric inflammation and oxidative stress, as well as ameliorates the colonic contractile dysfunctions. 

**Figure 6:**
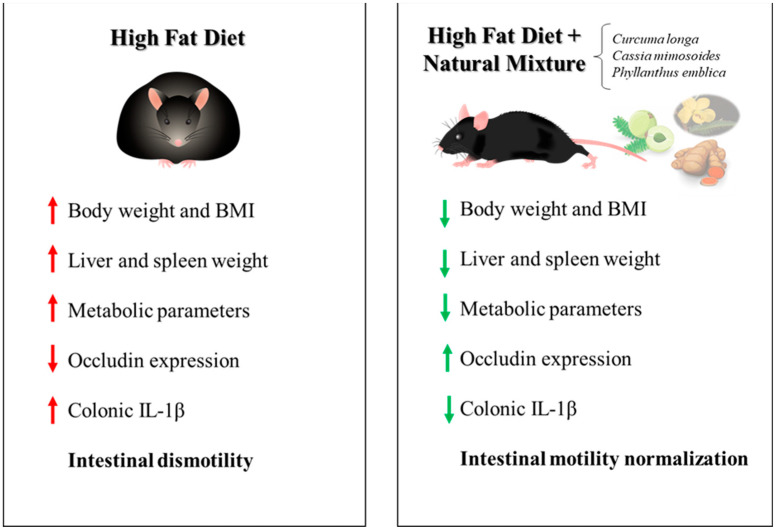
Schematic representation of the anti-obesity properties of dietary supplement, containing the dry extract of *Curcumin*, *Emblica* and *Cassia*, in a murine model of HFD diet.

To pursue these aims, we employed a murine model of HFD-induced obesity, which closely mimics a human obesity condition [10,11,17,18,28]. In our study, consistent with previous reports [10,11,17,18,28], obese mice displayed a marked increase in body weight, BMI and plasmatic MPO levels, along with significant alterations of systemic metabolic indices, such as blood total cholesterol, triglycerides and glycated hemoglobin levels (regarded as an indirect index of insulin resistance [29,30,31,32]), thus further confirming the suitability of HFD murine model. Moreover, HFD mice showed signs of enteric inflammation and neutrophil infiltration, along with an increase in spleen weight (index of chronic systemic inflammation and immune system activation [33]) and liver weight, which represent the prodromal steps leading to the development of hepatic steatosis [5,34,35]. In this setting, dietary supplementation with the natural mixture significantly counteracted the increase in body weight, BMI and metabolic parameters, along with neutrophil infiltration, as documented by a significant reduction in faecal calprotectin levels. Interestingly, the plant-based food supplement was also able to prevent the increase in spleen and liver weight. Of note, these ameliorative effects of the natural mixture may be ascribed to the ability of *Curcuma* L., *Cassia* M. and *P. emblica* to counteract body weight gain and oxidative stress associated with obesity, in accordance with previous studies [12,13,14]. In particular, *Curcuma* L. and *P. emblica* showed anti-obesity properties, inhibiting adipogenesis and regulating lipid metabolism, as well as preventing the increase in liver weight. In parallel, *Cassia* M., besides normalize metabolic parameters such as blood total cholesterol and triglycerides levels, displayed anti-oxidant properties, reducing the levels of reactive oxygen species (ROS) [12,13,14,36]. Our results corroborate interesting anti-obesity properties of such plant-based food supplements, preventing the increase in body, liver and spleen weight, as well as counteracting the metabolic modifications associated with a hypercaloric diet. It is well recognized that HFD consumption determines an impairment of the intestinal barrier structure, triggering a low-grade systemic inflammation and promoting the onset of a metabolic endotoxemia. In line with this view, we observed a reduction in the expression of occludin, one of the main tight junction proteins involved in preserving intestinal barrier integrity, along with an increase in circulating LPS binding protein (LBP) in obese mice, thus confirming changes in IEB structure and integrity following HFD intake. Of note, serum LPS is widely accepted as marker for the assessment of in vivo intestinal permeability; therefore, an increase in serum LPS levels has been associated with disorders displaying an increased intestinal permeability as common pathological feature, such as patients with inflammatory bowel diseases, irritable bowel syndrome, necrotizing enterocolitis or celiac disease [37,38,39,40]. In addition, HFD mice showed an increased expression of major inflammatory signals, such as TLR-4 and related downstream signaling molecules (MyD88 and NF-kB p65), which plays a critical role for a successful immune response [41]. Of note, the activation of TLR-4/MyD88/NF-κB pathways spur the expression of several pro-inflammatory cytokines, including IL-1β, that play pivotal roles in altering epithelial barrier integrity [24]. Interestingly, the administration of the dietary supplement significantly reinforced the IEB structure and integrity, as documented by a reduction in circulating LPS and normalization of tight junction occludin expression, as well as to counteract the increase in IL-1β levels in colonic tissues of obese mice. These ameliorative effects of such natural mixtures on intestinal barrier structure and inflammatory parameters are likely to be ascribed to the strengthening properties and anti-inflammatory abilities of *Curcuma* L. and *Cassia* M., respectively. Indeed, it has been demonstrated that the treatment with curcumin can significantly reduce the circulating LBP levels and improve IEB integrity by upregulating intestinal ZO-1, occludin and claudin-1 expression in murine models of sepsis, intestinal ischemia-reperfusion injury and type 2 diabetes mellitus [42,43,44]. In parallel, treatment with *Cassia mimosoides* normalized the inflammatory parameters, reducing circulating IL-1β and TNF levels in a HFD mouse model [36]. Such results highlight the strengthening effects on the IEB integrity and anti-inflammatory properties of this dietary supplement. The presence of an inflammatory condition in the gut, resulting from a weakening of the intestinal barrier, can alter digestive motility, triggering morphological and functional changes in the enteric neuromuscular compartment [45,46]. In this regard, changes in enteric tachykininergic pathways and a marked reduction in faecal output has been reported in several pre-clinical studies performed on HFD mice [10,17], corroborating the link between obesity, enteric inflammation and gut dysmotility. Of note, it is well known that an increase in colonic tachykininergic contractions, due to a marked substance P release, is associated with changes in peristaltic movements, resulting in a reduced colonic propulsive activity. In this respect, several epidemiological studies showed a high prevalence of enteric motor dysfunctions, including constipation and abdominal pain, in obese patients [47]. Consistent with this evidence, in the present study, we observed alterations of colonic excitatory neuromotility, characterized by an exalted tachykininergic pathway, in obese mice. Interestingly, dietary supplement induced a normalization of the abnormal tachykininergic contractions in HFD mice, similar to what was observed in animals fed with a standard diet, highlighting the ability of such natural mixtures to improve the bowel motor dysfunctions associated with obesity. Of note, this beneficial effect on colonic motility is likely to be ascribed to the anti-inflammatory properties of *Cassia mimosoides* and *Curcuma longa*. Indeed, it has been demonstrated that the presence of intestinal inflammation determines a reorganization of neurochemical coding on the enteric neurons, resulting in an increase in the tachykinergic contractions with consequent alteration of gut motility in obesity [10]. Therefore, the administration of natural products with anti-inflammatory properties could exert an ameliorative effect on colonic contractions due to their ability to curb the enteric inflammatory responses. In parallel, the restoration of tachykinergic contractions with natural mixtures could also be ascribed to the myorelaxant effects of *Cassia* M. and *Curcuma* L. on the mouse ileum and colon. Indeed, it has been described that treatment with *Cassia* and *Curcumin* is able to relieve the inflammatory-related intestinal dysmotility through a spasmolytic action on excitatory neurotransmitter pathways [48,49,50,51]. In conclusion, the present work provides evidence that dietary supplement, containing the dry extract of *Curcumin*, *Emblica* and *Cassia*, exerts interesting anti-obesity properties, preventing the increase in body, liver and spleen weight, as well as counteracting the metabolic alterations associated with a hypercaloric diet. In addition, such a natural mixture, besides exerting strengthening effects on the IEB integrity, shows anti-inflammatory properties and relieves the bowel dysmotility associated with obesity through a normalization of excitatory tachykininergic colonic contractions (Figure 6). In line with this view—however, more focused studies are needed—it is conceivable that a dietary supplementation with this natural mixture could also exert beneficial effects in other pathological conditions characterized by inflammatory-related intestinal dysmotility, such as inflammatory bowel disease or irritable bowel syndrome [52,53].

## Data Availability

The data presented in this study are contained within the article.
